# Cascadic failure and preferential decay via pruning mediated percolation on interdependent networks: implications for schizophrenia

**DOI:** 10.1192/j.eurpsy.2024.457

**Published:** 2024-08-27

**Authors:** K. Szalisznyó, P. Érdi, D. N. Silverstein

**Affiliations:** ^1^Psychiatry, Uppsala University, Department of Medical Sciences, Uppsala University Hospital, Uppsala, Sweden; ^2^Computational Sciences Department, Wigner Research Center for Physics, Budapest, Hungary; ^3^Department of Physics and Department of Psychology, Kalamazoo College in Kalamazoo, Kalamazoo, United States; ^4^Agora for Biosystems, Sigtuna foundation, Sigtuna, Sweden

## Abstract

**Introduction:**

During adolescence the brain is dynamically changing. Destabilization and acceleration of the normal adolescent synaptic pruning process is likely a contributing factor to the neuropathology of schizophrenia. Details on whether normal pruning effects weaker synapses more or uniformly all synapses with different strengths, needs to be further evaluated. Widespread impairment in structural connectivity in schizophrenic patients involving several cortical and subcortical areas, has been previously described. In this computational study, we investigated a stochastic percolation process in interdependent networks, motivated by pathological synaptic pruning. We examined preferential decay in the connectivity decremental process, as well as differential pruning in interconnected subnetworks. Finally, the speed of the percolation process, as well as the potential for pharmacological interventions of percolation in random networks was explored. Statistical structural properties of decaying networks pinpointed several network attributes which the disintegration and phase transitions qualitatively depended on.

**Objectives:**

The following objectives were explored: 1.) Apart from a random percolation process, we investigated preferential decay of the connections. We introduced different percolation rules for various connection types. 2.) Based on previous experimental results, we assumed that different interconnected neural subpopulations prune differently, therefore we explored differential pruning process in the subnetworks. 3.) The speed of the percolation was studied and the pharmacological synaptic connectivity change was also analyzed.

**Methods:**

We considered two inter-connected randomly connected networks, where the connections were removed during the percolation process. Simulations were partially performed using Octave on a Lenovo Thinkpad running the Linux operating system and partially performed on a supercomputer at UPPMAX (NAISS Small Compute 2023 Dnr: NAISS 2023/22-102).

**Results:**

We found that the coupled network system shows rich percolation behaviors with phase transitions for various coupling strength and coupling patterns. The phase transitions of both layers are altered qualitatively between discontinuous, mixed and continuous. Recursively developing percolation in interdependent networks can cause complete fragmentation of these networks, resulting in cascadic failure which might be related to schizophrenia symptoms.

**Conclusions:**

This computational study analyzes the pruning-mediated percolation in interdependent neural networks. Consequences of the pathological overpruning were related to the attributes of the interdependent network properties. Implications for schizophrenia development and predictions for compensatorial iatrogenic percolation was also pinpointed and discussed.

**Disclosure of Interest:**

None Declared

